# Structural evolution and strain induced mixing in Cu–Co composites studied by transmission electron microscopy and atom probe tomography

**DOI:** 10.1016/j.matchar.2014.12.022

**Published:** 2015-02

**Authors:** A. Bachmaier, H. Aboulfadl, M. Pfaff, F. Mücklich, C. Motz

**Affiliations:** a Chair of Materials Science and Methods, Saarland University, Saarbrücken, Germany; b Chair of Functional Materials, Saarland University, Saarbrücken, Germany; c INM-Leibniz Institute for New Materials, Saarbrücken, Germany

**Keywords:** High-pressure torsion, Atom probe tomography, Transmission electron microscopy, Cu–Co, Nano-composite, Microstructure, Mechanical alloying

## Abstract

A Cu–Co composite material is chosen as a model system to study structural evolution and phase formations during severe plastic deformation. The evolving microstructures as a function of the applied strain were characterized at the micro-, nano-, and atomic scale-levels by combining scanning electron microscopy and transmission electron microscopy including energy-filtered transmission electron microscopy and electron energy-loss spectroscopy. The amount of intermixing between the two phases at different strains was examined at the atomic scale using atom probe tomography as complimentary method. It is shown that Co particles are dissolved in the Cu matrix during severe plastic deformation to a remarkable extent and their size, number, and volume fraction were quantitatively determined during the deformation process. From the results, it can be concluded that supersaturated solid solutions up to 26 at.% Co in a *fcc* Cu–26 at.% Co alloy are obtained during deformation. However, the distribution of Co was found to be inhomogeneous even at the highest degree of investigated strain.

## Highlights

• Structural evolution in a deformed Cu–Co composite is studied on all length scales.
• Amount of intermixing is examined by atom-probe tomography.
• Supersaturated solid solutions up to 26 at.% Co in Cu are observed.

## 1 Introduction

Methods of severe plastic deformation, such as high-pressure torsion (HPT), are very powerful tools for introducing significant grain refinement in a wide range of initially coarse grained single phase materials and alloys [1–3]. Deformation of multiphase materials by HPT can lead to nanocomposites with an even smaller grain size in the order of ~ 10 nm [4]. In contrast to single phase materials and alloys, two or three phase materials deformed by HPT, whose microstructural evolution might be considerably different and dependent on its individual constituents, have not been intensively investigated [5,6]. Metastable solid solutions can form during deformations if the multiphase material consists of components exhibiting no equilibrium solid solubility. During ball milling, the phenomena of metastable and stable phase formation have been intensively investigated and explained by an interdiffusion reaction of the components for systems with a negative heat of mixing [7]. For alloys with positive heat of mixing, a diffusion reaction generally results in decomposition. Although often reported in recent years for different alloy systems with positive heat of mixing (Cu–Cr, Cu–W, Cu–Fe) deformed by HPT [8–14], the formation of metastable solid solutions is far from being well understood for ball milling and HPT.

In a recent paper, we have reported that the formation of supersaturated solid solutions during HPT of a Cu–Cr composite material, which is a combination of a soft and ductile matrix containing a hard and brittle second phase, is mainly controlled by a mechanical mixing mechanism and the amount of mixing is controlled by the strain level [14]. Mainly based on energy-dispersive synchrotron diffraction measurements, in which the domain size, the microstrain and the change of the lattice parameter of both phases as a function of the applied strain was evaluated, detailed investigation of the microstructural evolution leading to the strain induced mixing during the HPT deformation was lacking.

In this study, a similar type of composite consisting of a soft, ductile Cu matrix containing brittle Co particles is processed by HPT to understand the process of microstructural evolution during HPT deformation in detail. The binary Cu–Co system, similar to the Cu–Cr system, possesses a large positive heat of mixing [15], has two different crystal structures at room temperature, the *fcc* Cu and *hcp* Co structure, and the formation of non-equilibrium solid solutions have already been observed in this alloy system during ball milling [16,17]. The deformation strain can be directly calculated and linked to the microstructural observations. Microstructural characterization was conducted on all length scales. First of all, extensive scanning electron microscopy (SEM) investigations including image analysis to quantitatively determine the size, numbers, and volume fractions of the Co particles and the microstructural evolution of the Cu matrix at different strains have been performed. Furthermore, transmission electron microscopy (TEM) investigations have been conducted to characterize the as-deformed microstructures. Analytical TEM investigations included energy-filtered transmission electron microscopy (EFTEM) and electron energy-loss spectroscopy (EELS). With EFTEM imaging, Co and Cu compositional maps were obtained. Quantification of EELS spectrum images in both phases and across Co and Cu phase boundaries yields information on the chemistry and amount of intermixing between Cu and Co phases with high spatial resolution. Atom probe tomography (APT) investigations were used to precisely determine the amount of intermixing at different levels of strain. APT is a suitable technique for our case here since it offers uniquely high spatial resolution in three-dimensions for identifying local composition distribution and interfacial mixing at the atomic scale [18–20]. Additionally to the microstructural characterization described above, the evolution of the microstructure was examined by hardness measurements.

By using the example of a simple Cu–Co model composite material, this work has two major aims: describing the microstructural evolution leading to grain refinement of the composite material and dissolution of the Co particles in detail and to confirm the formation of supersaturated solid solutions in this system during HPT.

## 2 Materials and methods

### 2.1 Initial material and HPT processing

The investigated material is a two-phase Cu–Co material containing about 26 at.% Co fabricated by RHP-Technology (Seibersdorf, Austria). Co powders (purity 99.8%) and Cu powders (purity 99.9%) were mixed and subsequently precompacted into samples with cylindrical shape. For comparison purposes, pure Cu powder samples (with the same purity) were also compacted. The hardness of the initial, compacted material was measured by Vickers microhardness using a load of 500 g (H_V0.5_). The individual hardness of both phases of the Cu–Co material were measured with a nanoindenter (ASMEC UNAT-SEM2) fitted with a Berkovich Indenter. Vickers hardness is calculated using the measured indentation hardness (20 indentations in each individual phase using a load of 2 mN). Conversion into conventional Vickers hardness is done using the InspectorX software package, whereby the difference between indentation and Vickers hardness is below 10% [21].

Processing of disk shaped samples by HPT deformation was conducted at room temperature using an HPT facility of the type described in earlier work [22]. The HPT processing was conducted under a pressure of 5 GPa with a rotation speed of 0.2 rpm. The disk samples (diameter 8.0 mm, thickness ~ 0.6 mm) were processed for five different numbers of revolutions (N = 1, 5, 7, 25 and 100 turns). All strains quoted in this study are given as von Mises equivalent shear strain ε_eq._ It is calculated according to [23]

(1) $$\varepsilon_{\mathrm{eq}}=\frac{2*\pi *r*N}{\sqrt{3}*t}\text{.}$$

In this equation, *N* is the number of applied rotations during HPT, *r* is the distance from the center of the sample and *t* is the sample thickness. In Table 1 an overview of the investigated strains and conducted experiments (microhardness measurements, microstructural characterization) at each strain is given.

### 2.2 Hardness measurements

Vickers microhardness measurements (H_V0.5_) were performed on deformed samples (N = 1, 5, 25 and 100) as function of the equivalent strain ε_eq_. Indents were made across the radii of the disk with a distance of 0.25 mm between the measurement positions and mean values of three individual indents at each position were determined. At selected positions, nanoindentation experiments were conducted with a cube corner diamond tip on a Hysitron TriboIndenter® with the Performech™ controller in displacement controlled mode with constant loading, hold and un-loading time (maximum displacement set to 50 nm). Several individual indents were made following a quadratic square (10 × 10 or 12 × 12 indents) with 0.7 μm distance spacing between each separate indent. All of the measured nanoindentation values were then used to construct color-coded contour maps at the selected positions over a total area of 49.0 μm^2^ or 70.6 μm^2^, respectively. The principal goal of the nanoindentation measurements was to provide a qualitative and visual presentation of the hardness distributions at different deformation strains at a smaller scale and not to compare the measured indentation hardness to Vickers microhardness.

### 2.3 Microstructural characterization

Microstructural examination was carried out by SEM using a Zeiss SIGMA™-VP field emission scanning electron microscope device equipped with an energy dispersive spectroscopy (EDS) detector. The microanalysis data was evaluated using the AZtec software (Oxford Instruments). All SEM micrographs were taken in tangential direction at selected positions (i.e. different equivalent strain ε_eq_) across the radii of the HPT deformed samples. Grain sizes were determined from back-scattered electron SEM micrographs with a high magnification at selected positions with the linear intercept method using the software Lince 2.4.2e [24]. To study the process of fragmentation and microstructural evolution of the Co phase as a function of the equivalent strain ε_eq_, the software program ImageJ was employed [25]. At each investigated position (i.e. equivalent strain ε_eq_), five separate back-scattered electron SEM micrographs are recorded and investigated (see Table 1 for the exact positions). The software automatically determines different phase parameters (such as a size, aspect ratio and area fraction) from the SEM images. The total number of Co particles per area (μm^2^) evaluated at each investigated position ranged from over 0.17 particles per μm^2^ at low strains to 0.008 particles per μm^2^ at a ε_eq_ of 446 (Table 2).

Additionally, TEM investigations were performed on a JEOL JEM 2010 instrument operated at 200 kV at a ε_eq_ = 98 and ε_eq_ = 446 in tangential direction. Selected area diffraction (SAD) patterns were recorded using a camera length of 30 cm. Thin lamellae for TEM analysis were prepared with a dual-beam focused ion beam/SEM workstation (Helios NanoLab 600™ from FEI) using a lift out method. Before lift out, an electron beam induced Pt-cap layer was deposited to provide protection from Ga implantation during specimen preparation. After lift out, the lamellae are then further thinned for electron transparency. The thinning was done with 30 kV of accelerating voltage, followed by thinning with 5 kV of accelerating voltage to minimize any possible preparation artifact.

X-ray diffraction (XRD) measurements for phase analysis were performed with a PANalytical X'pert diffractometer in a Θ/2Θ geometry using Cu-Kα radiation on the initial, compacted material and on the HPT deformed sample (N = 100).

Analytical TEM investigations including scanning TEM (STEM), EFTEM and EELS were used to characterize the microstructure of the deformed material at lower deformation strain (ε_eq_ = 102) in more detail. STEM, EFTEM and EELS studies were carried out using a cold field emission gun TEM/STEM (JEOL JEM-ARM 200 F) at 200 keV. It is equipped with a STEM Cs corrector (CESCOR; CEOS GmbH Heidelberg) and a post-column EELS spectrometer (GIF QuantumER™ from Gatan). For STEM, the annular dark-field (ADF) detector was used with a camera length of 8 cm, resulting in a collection angle range of 70–280 mrad. The EELS measurements were carried out with a beam current of 530 pA, a collection semi-angle of 10.4 mrad, a spectrometer entrance aperture of 2.5 mm and a dispersion of 0.25 eV/ch. EFTEM images were acquired with the DigitalMicrograph® software from Gatan using the three-window method at the Cu L edge (931 eV) and the Co L edge (779 eV). The exposure time was 5 s with a binning of 4. A drift correction was applied to enhance the image quality.

The TEM sample preparation for the STEM, EFTEM and EELS studies included the following steps: Disks were cut at a radius of 2.5 mm (ε_eq_ = 102), mechanically thinned and polished to a thickness of about 100 μm from HPT samples deformed for 7 rotations. Next, mechanical dimpling until the thinnest part reached a thickness of about 10 μm was conducted. Then, the samples were ion-milled with Ar ions at 4–5 kV under an incidence angle of 6° using a Gatan Precision Ion Polishing System until perforation was obtained. Due to conventional TEM preparation in this case, the electron beam was perpendicular to the shear plane of the disks for all STEM, EFTEM and EELS investigations shown in this work.

APT was used to study the local composition at the same positions investigated by TEM (ε_eq_ = 98 and ε_eq_ = 446). Site specific sample preparations were done in a dual-beam focused ion beam/SEM workstation implementing the in-situ lift out technique [26]. A 200 nm thick electron beam Pt-cap layer was first deposited by physical vapor deposition for reducing Ga implantation during specimen preparation. 2 kV was used for final steps during specimen shaping to minimize Ga induced damage.

The measurements were carried out using a LEAP™ 3000X HR CAMECA™ system. The specimens were measured in laser pulsing mode (532 nm wavelength) with a repetition rate of 200 kHz, a temperature of 60 K and laser pulse energy of 0.4 nJ. The data were reconstructed using the standard algorithm developed by Bas et al. [27] and analyzed with the software CAMECA^TM^ IVAS 3.6.6. By measuring the charge-state ratios of 63Cu^+^/63Cu^2 +^ and 59Co^+^/59Co^2 +^, Kingham diagrams were used to estimate the field strength values resulting in a field of ~ 28 V/nm for the single phase Cu–Co alloy phase regions [28]. The compositional distribution in the datasets was analyzed by combining frequency distribution functions, 2D composition maps and linear compositional profiles [29].

## 3 Results

### 3.1 Hardness and microstructural evolution as function of the applied strain

**Fig. 1:**
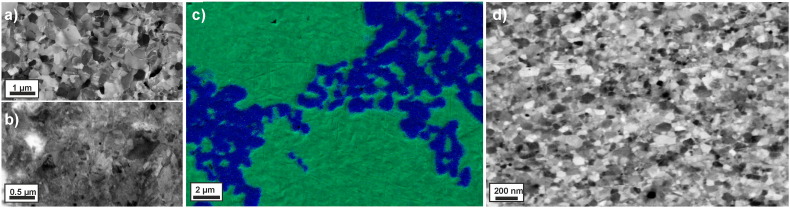
Back-scattered electron micrographs illustrating the microstructure of the Cu phase (a) and Co phase (b). (c) Initial microstructure of the material: SEM micrograph superimposed by an EDS map where all regions belonging to Co are marked as blue and all regions belonging to Cu are green. (d) Back-scattered electron micrographs of the microstructure of the Cu–Co alloy after HPT deformation to a strain of 446.

**Fig. 2:**
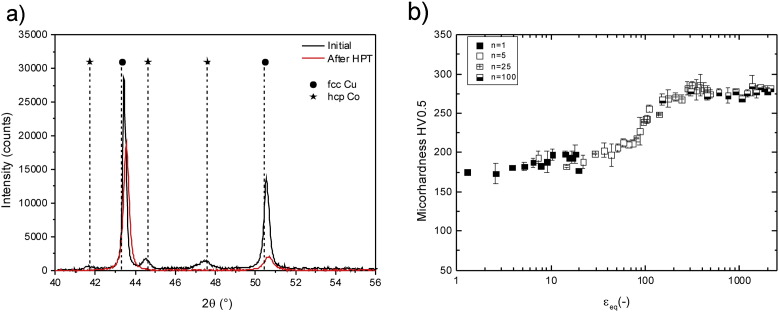
(a) XRD patterns recorded in the initial condition and after HPT deformation for 100 rotations. (b) Evolution of microhardness plotted as a function of the equivalent strain ε_eq_ for Cu–Co samples deformed for N = 1, 5, 25, 100 revolutions.

In Fig. 1(a) and (b), the initial microstructure of the Co and Cu phases in the Cu–Co material is shown. The microhardness of the undeformed, as-fabricated Cu–Co sample is 137 ± 39 HV_._ The grain size in the Cu phase is 486 ± 110 nm, and the grain size of the Co phase is significantly smaller (about 100 nm) with only single grains having a grain size above the nanometer range. XRD investigation of the initial, un-deformed material confirms its two-phase nature, consisting of two distinct *hcp* Co and *fcc* Cu phases (Fig. 2(a)). In the SEM image shown in Fig. 1(c), the phase distribution of *hcp* Co and *fcc* Cu in the initial condition is illustrated with a full-color overlay showing the variations in the EDS spectrum. Regions appearing green in the micrograph represent the Cu phase, regions appearing blue consist of the Co phase. It is apparent that the Co phase is inhomogeneously distributed and clustered. The individual hardness of the Cu phase is 103 ± 12 HV in the initial condition. Due to its nanocrystalline grain size, the hardness of the Co phase is very high (394 ± 21 HV). Hence, a very hard second phase (Co) is embedded in a relatively soft matrix phase (Cu), similar as in a classical composite material. In the following, the Cu–Co material is treated as if it is such a composite material consisting of a Cu matrix containing hard Co particles.

**Fig. 3:**
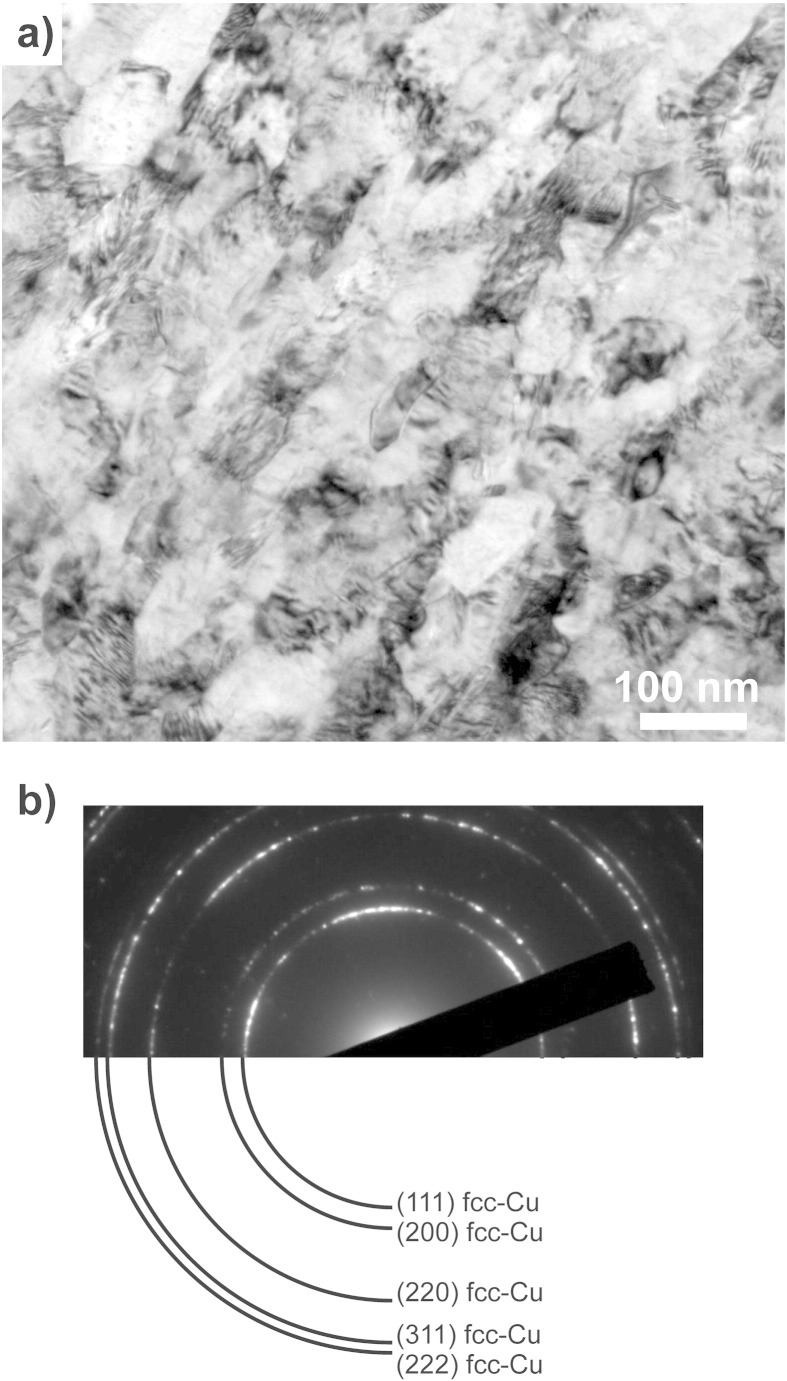
TEM bright field image showing the microstructure of the Cu–Co material at an equivalent strain of 446 (a) and corresponding SAD pattern (b). Only Deybe–Scherrer rings of *fcc* Cu are indicated in the SAD pattern.

Processing the Cu–Co sample by HPT leads to strong grain refinement in the Cu phase and an increase in the overall hardness of the deformed material. The microstructure of the sample is homogeneously refined in the steady state region. This is confirmed by SEM investigations recorded at various positions in this region, which correspond to a saturation of the microstructural refinement and in which application of further HPT deformation will not induce further grain refinement. As an example, the microstructure at equivalent strains ε_eq_ of 446 are shown in Fig. 1(d). Compared to the initial state, an ultrafine grained structure with a mean grain size of 101 ± 20 nm is visible. The microstructure in the steady state is additionally investigated by TEM. Fig. 3(a) shows a bright-field TEM micrograph, which was recorded at an equivalent strain of ε_eq_ = 446. It illustrates an ultrafine grained microstructure with well-defined grains, the majority having a size around 100 nm. Most of the grains are elongated in the shearing direction, but some smaller, truly nanocrystalline, equiaxed grains are also visible. Contrast variations inside the grains indicate a huge amount of defects in the as-deformed structure. In the SAD pattern shown in Fig. 3(b), only *fcc* Cu Debye–Scherrer rings of (*111*), (*200*), (*220*), (*311*) and (*222*) are observed. The SAD pattern is consistent with observations from XRD measurements on the as-deformed Cu–Co material. Contrary to the initial state, only *fcc* Cu peaks, which are slightly broadened and shifted to higher angles, are detected in the XRD pattern (Fig. 2a).

**Fig. 4:**
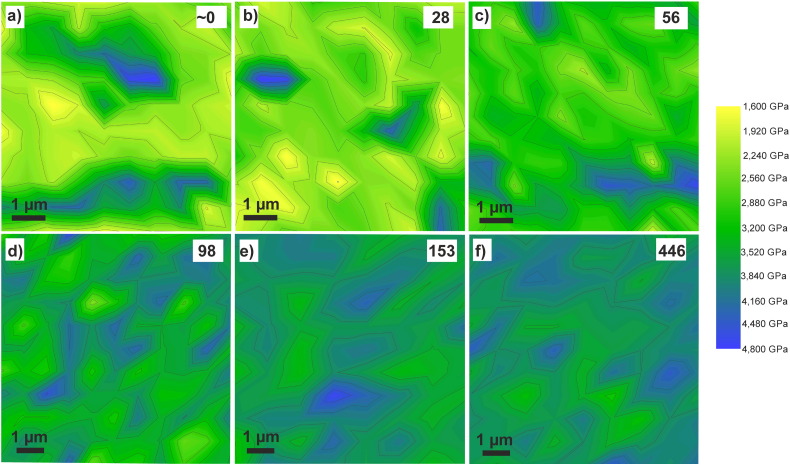
Two-dimensional color-coded contour maps of the hardness distributions at different positions (i.e. equivalent strain ε_eq_) of the HPT disk: (a) ε_eq_ ~ 0, (b) ε_eq_ = 28, (c) ε_eq_ = 56, (d) ε_eq_ = 98_,_ (e) ε_eq_ = 153 and (f) ε_eq_ = 446. The size of the maps shown in (a) to (c) is 7.0 × 7.0 μm^2^, the size of the map shown in (d) to (f) is 8.4 × 8.4 μm^2^. The indentation hardness values are given in GPa in the figure legend (only qualitative purpose).

A plot of the microhardness as a function of the applied strain illustrates the hardness evolution with increasing deformation strain in detail (Fig. 2(b)). The hardness increases almost instantly from about 140 HV to about 175 HV in the beginning of the HPT deformation. A further slight hardness increase to ~ 210 HV is visible up to an equivalent strain of 10. Between strains of 10–90, no significant change in the hardness is visible. Thereafter, a strong hardness increase to 270 HV, in a strain range from 90 to 150, is measured. At higher strains, the microhardness remains nearly constant and a “second” steady state of the microhardness is reached. In Fig. 4, two-dimensional color-coded contour maps, illustrating the indentation hardness distributions for strains of (a) ε_eq_ ~ 0, (b) ε_eq_ = 28, (c) ε_eq_ = 56, (d) ε_eq_ = 98, (e) ε_eq_ = 153 and (f) ε_eq_ = 446, are shown. The indentation hardness values (GPa) are presented by unique colors given at the right in the figure. It is apparent that high indentation hardness differences are recorded at a strain of ~ 0, which illustrates the distribution of the Co particles in the Cu matrix (Fig. 4(a)). The hardness ratio between the highest and the lowest measured indentation hardness is 2.9 at a strain of ~ 0. At low deformation strain, regions with lower indentation hardness decrease in size. At a strain of 56, only very small regions exhibit an indentation hardness lower than 2 GPa (Fig. 4(c)). Additional deformation to a strain of 98 reduces the indentation hardness differences even more (Fig. 4(d)). For strains > 150, a rather homogeneous high indentation hardness throughout the total investigated areas is visible (Fig. 4(e) and (f)).

**Fig. 5:**
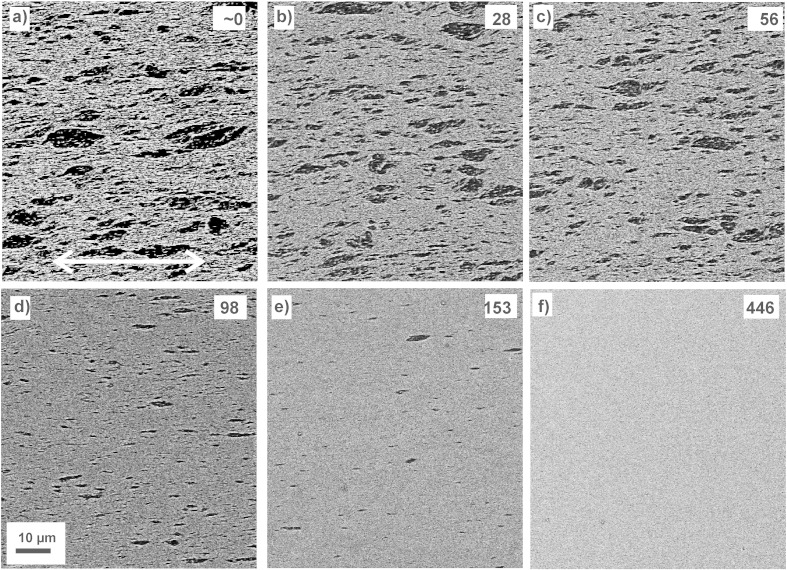
Back-scattered electron micrographs taken at different positions (i.e. equivalent strain ε_eq_): (a) ε_eq_ ~ 0, (b) ε_eq_ = 28, (c) ε_eq_ = 56, (d) ε_eq_ = 98_,_ (e) ε_eq_ = 153 and (f) ε_eq_ = 446. The Co particles appear dark in the micrographs. The magnification and the shearing direction are the same in all micrographs.

**Fig. 6:**
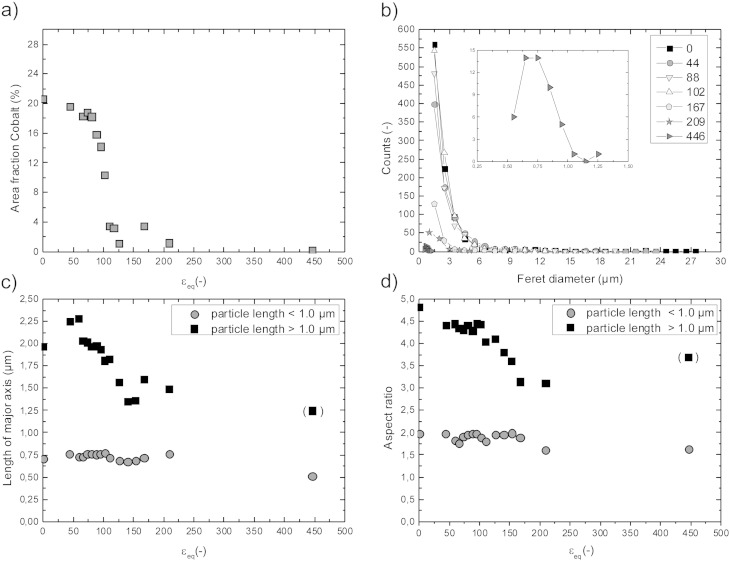
(a) Area fraction of the Co phase as a function of the equivalent strain ε_eq_. (b) Particle size distribution at different strains (ε_eq_ = 0, 44, 88, 102, 167, 209 and 446). (c) Length of the major axis of an ellipse fitted to the particles as a function of the equivalent strain ε_eq_. (d) Aspect ratio (length of major axis/length of minor axis) of the Co particles as a function of the equivalent strain ε_eq._ In (c) and (d), particles are divided into two groups (particles with a length > 1 μm and particles with a length < 1 μm). The values given in brackets in (c) and (d) are mean values determined from only 3 particles with a length > 1 μm, which have been included for the sake of completeness (compare Table 2).

In Fig. 5, SEM micrographs with a lower magnification are shown to illustrate the microstructural evolution of the Co particles in the Cu matrix with increasing deformation. The shearing direction lies along the horizontal direction of all micrographs, which is indicated in Fig. 5(a). Co and Cu phases can be easily differentiated by their brightness levels — Cu regions are brighter, Co regions appear darker — in the back-scatter electron micrographs. At strains of 0 to 153 (Fig. 5(a)–(e)), a rather inhomogeneous microstructure is observed. Some large, but also very small Co particles are visible in the Cu matrix. The particles, which seem to exhibit a slight elongation in the shearing direction, are randomly distributed in the Cu matrix. With increasing strain level, their overall size is refined and their density continuously decreases. In the micrograph recorded at an equivalent strain of ε_eq_ = 446, the microstructure appears to be free of micro-particles (Fig. 5(f)). SEM analysis with higher magnification carried out at this position reveals that even at this amount of applied strain, single Co particles can be still detected in the micrographs. The process of fragmentation and microstructural evolution of the Co particles is quantitatively investigated in detail as a function of the equivalent strain ε_eq_ with the image analysis software. In Fig. 6(a), the area fraction of the Co phase as a function of the equivalent strain ε_eq_ is plotted. At low strains (0 < ε_eq_ < 80), the area fraction of the Co phase decreases only slightly from 21 to 18%. Between a strain of 80 and 110, which is about one third more strain, the area fraction of the Co phase reduces quickly to 3%. At the highest amount of strain, the area fraction of the Co phase is only 0.2%. As an example of the evolution of the Co particle size during deformation, particle size distributions at different investigated strains (ε_eq_ = 0, 44, 88, 102, 167, 209 and 446) are shown in Fig. 6(b). As particle size, the Feret diameter, which is the longest distance between any two points along the selection boundary of a single Co particle, was determined by the software. In Fig. 6(b), particles with a Feret diameter below 0.5 μm are excluded from the analysis. In general, it cannot be excluded that particles with a size smaller than 0.33 × 0.14 μm^2^, the smallest of all detected particles, still exist in the material, which are not detected by the image analysis software at the used magnification. In Fig. 6(c), the lengths of the major axis of ellipses fitted to the Co particles as a function of the equivalent strain ε_eq_ are plotted. In Fig. 6(c) and (d), the Co particles are divided into two groups: Particles with a length of their major axis > 1 μm, designated as “large” particles in the following, and particles with a length of their major axis < 1 μm, are considered separately. The length of the large Co particles increases at low deformation strains from 2.0 to 2.3 μm at a ε_eq_ of 59. Between strains of 59 to 95, the length of the large particles decreases slowly to 1.9 μm. At higher strains (95 < ε_eq_ < 126), the length of the large particles is quickly reduced to 1.6 μm. The same trend is reflected in the number of particles per area (μm^2^) with a length larger than 1 μm, it decreases from 0.14 to only 0.0004 Co particles per μm^2^ at the highest amount of strain (Table 2). On the contrary, the length of the particles with a major axis < 1 μm remains nearly constant during the whole applied strain regime. Nevertheless, their number per area reduces as well from 0.03 to 0.01 at a strain of 446 (Table 2). Finally, the aspect ratio (length of major axis divided by length of minor axis) as a function of the equivalent strain ε_eq_ is plotted in Fig. 6(d). The aspect ratio of the “large” Co-particles varies between 4.8 and 4.4 in the beginning of the deformation (0 < ε_eq_ < 100). Afterwards, the aspect ratio continuously decreases to 3.1 at higher strains. The aspect ratio of the small Co particles stays rather constant as a function of strain.

**Fig. 7:**
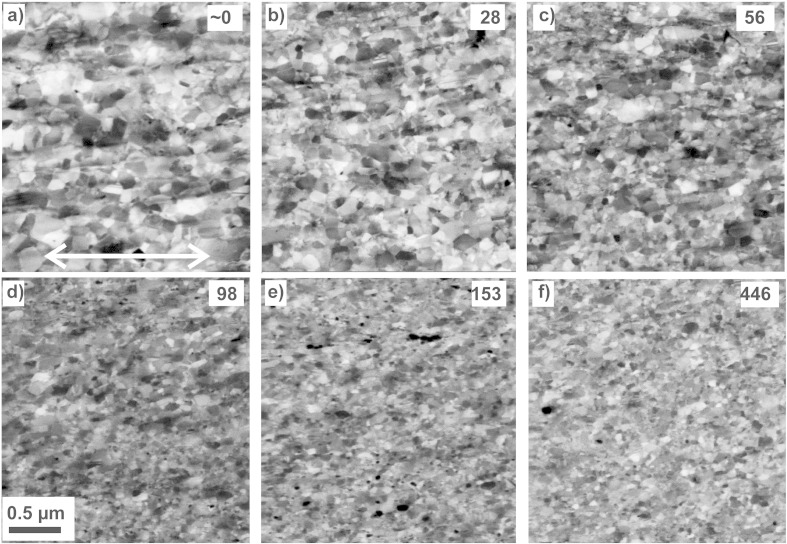
Back-scattered electron micrographs taken at different positions (i.e. equivalent strain ε_eq_) illustrating the structural evolution of the Cu matrix: (a) ε_eq_ ~ 0, (b) ε_eq_ = 28, (c) ε_eq_ = 56, (d) ε_eq_ = 98_,_ (e) ε_eq_ = 153 and (f) ε_eq_ = 446. The magnification and the shearing direction is the same in all micrographs.

To illustrate the microstructural evolution of the Cu matrix in detail, SEM micrographs showing predominantly the Cu phase were recorded at the same positions as in Fig. 5. In Fig. 7, the microstructure of the Cu matrix deformed to different strains of (a) ~ 0, (b) 28, (c) 56, (d) 98, (e) 153 and (f) 446 is shown. Even at a strain of ε_eq_ ~ 0, an ultrafine grained structure in Cu has already developed. Compared to the initial microstructure of the Cu phase (shown in Fig. 1(a)), the microstructure is refined even at this small amount of applied strain. The Cu grains are furthermore slightly elongated in the shearing direction, which is along the horizontal direction of all micrographs in Fig. 7. Due to a very fine microstructure, which is visible between the large grains, a quantitative determination of the grain size from back-scattered electron micrographs has not been performed at medium strains. Qualitatively, no significant change in the microstructure is visible to a strain of 56 (Fig. 7(c)). The size of the large grains is decreasing to a minor extent, the area of the fine microstructure is simultaneously increasing. At strains higher than 98, significant further grain refinement occurs (Fig. 7(d)). At an equivalent strain of ε_eq_ = 153 and ε_eq_ = 446, no difference in the observed microstructures is visible (Fig. 7(e)–(f)). A “second” steady state in the microstructural refinement with a significant smaller grain size compared to the first steady state is reached. The microstructural change is consistent with the hardness change. The qualitative impression of a reduced grain size is further confirmed by grain size measurements from back-scattered electron micrographs at these positions. At an equivalent strain of ε_eq_ = 153 and ε_eq_ = 446, a mean grain size of 102 ± 11 nm and 101 ± 20 nm is measured, respectively.

**Fig. 8:**
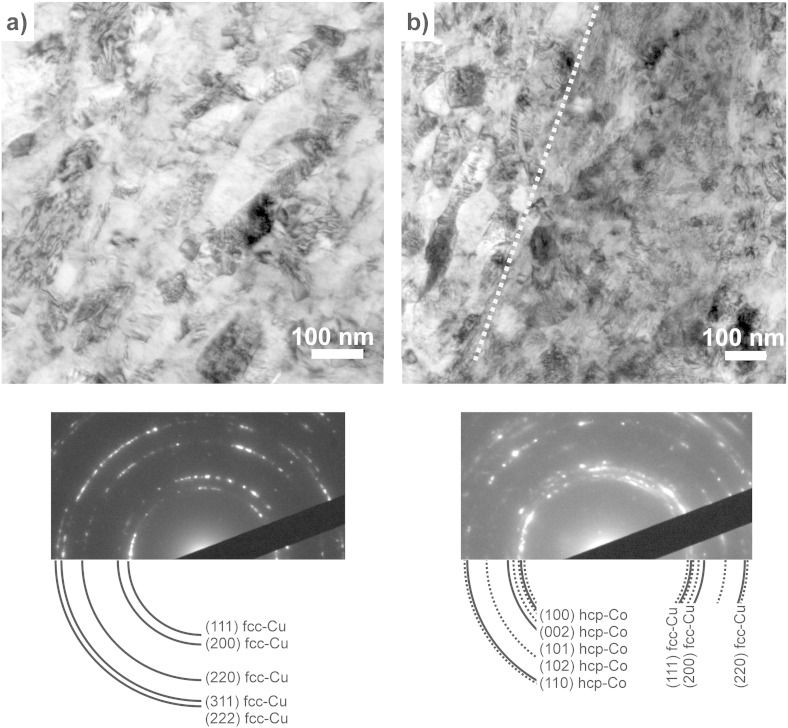
TEM bright field images showing the microstructure of the Cu–Co material at an equivalent strain of 98 in a homogeneous Cu phase region (a) and at the transition between a Cu phase and a Co phase. Corresponding SAD pattern are shown as insets. Only Deybe–Scherrer rings of *fcc* Cu are indicated in (a), Debye–Scherrer rings of *fcc* Cu and hcp Co are indicated in (b).

The microstructure at a strain of 98 is additionally investigated by TEM. Fig. 8(a) shows a bright-field TEM micrograph, which was recorded in a homogeneous Cu region in between the Co particles. It illustrates an ultrafine grained microstructure with well-defined grains, most of the grains are elongated in the shearing direction as well. The majority of the grains exhibit a size above 100 nm. Compared to the microstructure in the “steady state” at a strain of 446, the microstructure is slightly coarser. In the corresponding SAD pattern, only *fcc* Cu Debye–Scherrer rings of (*111*), (*200*), (*220*), (*311*) and (*222*) are observed. Fig. 8(b) shows a bright-field TEM micrograph recorded in a two-phase region. The transition between the Co particle and the Cu matrix is schematically indicated by the dashed white line. In the Co phase, a nanocrystalline structure, with huge contrast variations inside the grains due to a huge amount of defects, is visible. The corresponding SAD pattern illustrates Debye–Scherrer rings of the *fcc* Cu phase as well as the *hcp* Co phase.

### 3.2 Analytical transmission electron microscopy

**Fig. 9:**
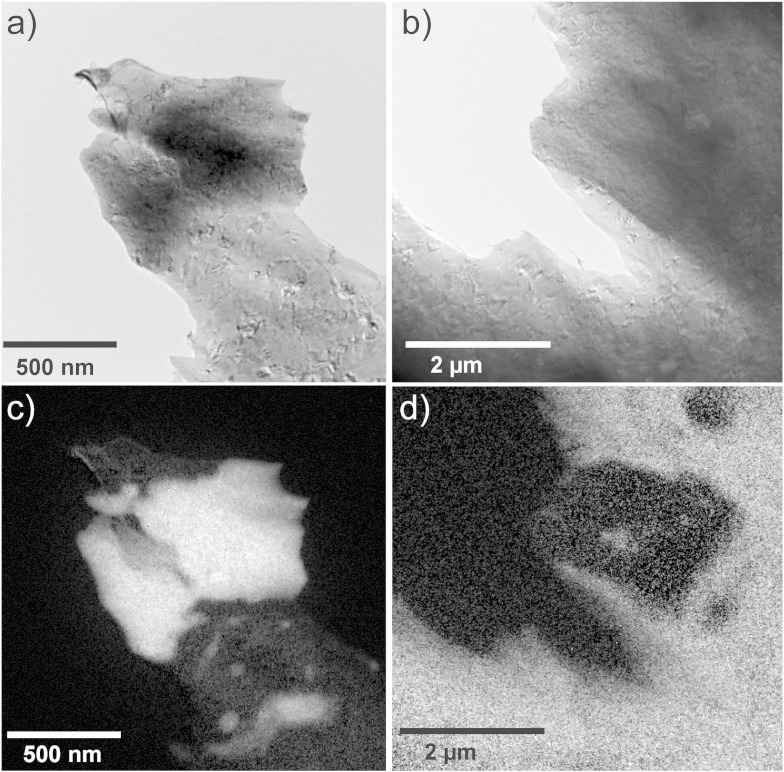
Distribution of Cu and Co phases after HPT processing to ε_eq_ of 102: EFTEM zero loss images (a, b) and corresponding energy filtered Co mapping (c) and Cu mapping (d).

**Fig. 10:**
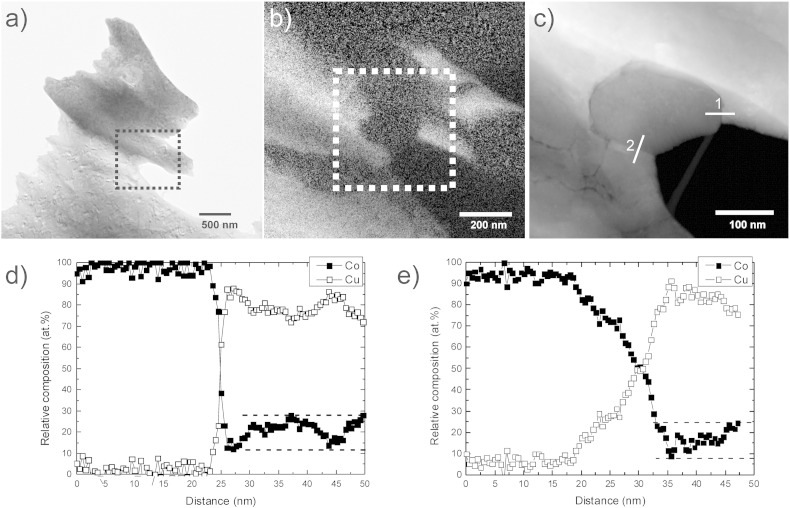
(a) EFTEM zero loss image, (b) energy filtered Cu mapping of the position indicated in (a) and (c) ADF STEM image of the position indicated in (b) with a higher magnification. EELS concentration profiles in atomic percent for Cu and Co along the line “1” (d) and line “2” (e) drawn in (c).

In Fig. 9(a) and (b), zero loss filtered images and corresponding core-loss energy filtered Co (c) and Cu mappings (d) are shown, which qualitatively illustrate the distribution of Cu and Co phases after HPT processing to a ε_eq_ of 102. To avoid grain overlap, thin edge regions of the samples were imaged and analyzed. In both images, Co particles embedded in a Cu matrix are apparent. Inside both particles, Cu-rich regions are visible. Furthermore, smaller Co fragments and small Co particles with size below 100 nm are apparent in the Cu matrix. In Fig. 10(a), a zero loss filtered image at the same deformation strain is shown, in which a “large” Co particle on the edge of the sample is imaged. In Fig. 10(b), an energy filtered Cu map with a higher magnification of the position indicated in (a) illustrates Cu and Co-rich regions in this area. The ADF STEM image, which is illustrated in Fig. 10(c), is positioned in such a way that a Co grain next to two Cu grains with a higher magnification is visible. EELS analysis was carried out to determine the local chemical distribution of Cu and Co at this position, which is shown in the concentration profiles obtained along line 1 and line 2 across the interphase boundaries marked in the STEM image (Fig. 10(d) and (e)). For the concentration profile along lines 1 and 2, 95 and 100 measurements with 0.5 nm spacing were conducted, respectively. In both composition profiles, Cu and Co phases are visible and nearly no Cu can be detected in the Co phase on the left side of the line scan in both cases. Along line 1, a Cu phase containing 12–28 at.% Co is subsequently measured. The concentration gradient along the line is quite small. Along line 2, a Cu phase in which 8–24 at.% Co is dissolved can be seen next to the Co phase. Although the scan is conducted at the edge region of the sample, the concentration gradient at the Co/Cu interface along line 2 seems to be affected by the overlapping of the Cu and Co grains. However, the data strongly suggests, that a supersaturated solid solution of Co in *fcc* Cu is formed during HPT processing, whereas nearly no Cu is dissolved in the Co phase.

### 3.3 Atom probe tomography

Based on TEM investigations, the formation of supersaturated solid solutions with a single phase *fcc* Cu structure is supposed. In the TEM-based analysis, the EELS signal is averaged over the thickness of the sample which is always a potential source of error. Hence, further correlative investigations using APT have been conducted at this strain to confirm and to measure the chemical distribution at a finer scale for Co in the Cu matrix. For evaluating the amount of mechanical intermixing at a higher deformation degree, the microstructural evolution at a strain of 446 (ε_eq_446) was additionally investigated in detail by APT. Site specific APT specimens at a strain of 98 were prepared from an homogeneous Cu single phase region (ε_eq_98-I), from a Cu-rich and Co-rich region (ε_eq_98-II) and directly from a Co particle (ε_eq_98-III) (see Supplementary Fig. S1).

**Fig. 11:**
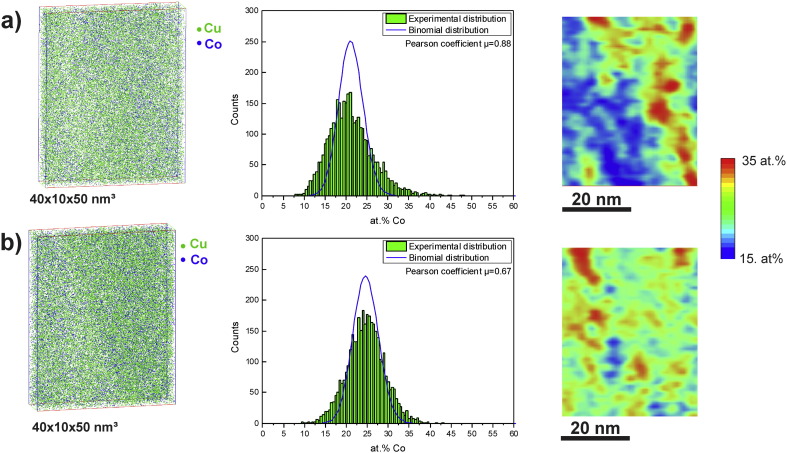
3D reconstructed sub-volumes and 2D composition maps of Cu–Co alloy samples deformed to a strain of 98 and 446: (a) ε_eq_98-I and (b) ε_eq_446. Cu and Co atoms are displayed in green and blue within the volume, respectively. The corresponding Co frequency distributions from the same sub-volumes compared to a random distribution are also shown in (a) and (b). The Pearson coefficient calculated for the given Co frequency distributions and the random distribution is also displayed. Concentration of Co (in at. %) in the 2D composition maps varies with color scale from 15 at.% (blue) to 35 at.% (red) as indicated on the right side.

**Fig. 12:**
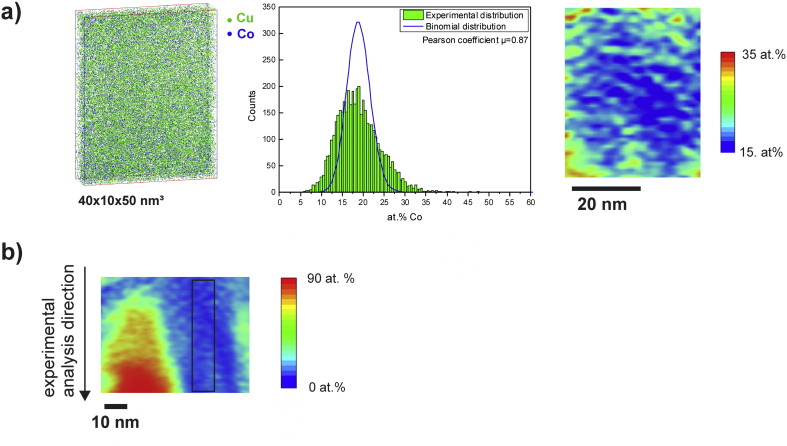
(a) 3D reconstructed sub-volume and 2D composition map of a Cu–Co alloy sample deformed to a strain of 98 (ε_eq_98-II) and (b) through-thickness representation (2D composition map) displaying the position of the sub-volume shown in (a). Cu and Co atoms are displayed in green and blue within the volume, respectively. The corresponding Co frequency distributions from the same sub-volume compared to a random distribution, the Pearson coefficient calculated for the given Co frequency distribution and the random distribution is also displayed. Concentration of Co (in at. %) in the 2D composition maps varies with color scale as indicated on the right side of the corresponding figures.

Figs. 11 and 12 show 3D reconstructed sub-volumes from the homogeneous regions of the Cu–Co alloy samples deformed to both strains (ε_eq_98-I (Fig. 11(a)), ε_eq_98-II (Fig. 12(a)) and ε_eq_446 (Fig. 11(b)). In all 3D reconstructed sub-volumes, Cu atoms are displayed in green and Co atoms are illustrated in blue. In Fig. 12(b), a 2D composition map illustrating the distribution of Co in a larger reconstructed cross-section of ε_eq_98-II is shown. The Co concentration is given in at.% indicated according to color (0 = blue; 90 = red). The selected sub-volume displayed in Fig. 12(a) is taken from the Cu-rich region (small boxes inserted in (b)). Considering the analyzed volumes illustrated in Figs. 11 and 12, the distribution of Co atoms in the Cu matrix seems to be fairly homogeneous and no spatial correlations in the distribution of the Co atoms are visually apparent at both positions and strains. The local compositions, determined in the displayed subvolumes in Fig. 11, is 23.9 ± 0.1 at.% Co and 76.0 ± 0.1 at.% Cu for (a) and 25.6 ± 0.1 at.% Co and 74.2 ± 0.1 at.% Cu for (b). The local composition of the subvolume displayed in Fig. 12(a) is 21.7 ± 0.1 at.% Co and 78.1 ± 0.1 at.% Cu. Hence, the agreement with the nominal composition of the alloy (74 at.% Cu, 26 at.% Co) is quite good.

Additionally, the frequency distributions of Co concentration are presented in comparison to the theoretical binominal distribution for random solid solution to investigate the degree of homogenization of Co in the Cu matrix for each sub-volume. Each dataset has the same size (40 × 10 × 50 nm^3^) and is divided into equal blocks of 200 atoms. In Figs. 11(a) and 12(a), the experimental distribution is widened to both sides and does not fit the random distribution, indicating that the Cu–Co supersaturated solid solution is not homogeneous in both cases. The experimental distribution shown in Fig. 11(b) deviates less from random compared to those at a strain of 98, which reveals less Co clustering effects in the analyzed dataset. For a more quantitative interpretation of the frequency distributions, the Pearson coefficient μ is determined for each dataset [30–32], which is 0.88 for ε_eq_98-I (Fig. 11(a)), 0.87 for ε_eq_98-2 (Fig. 12(a)) and 0.67 for ε_eq_446 (Fig. 11(b)). The calculated μ reflects the difference between the datasets obtained at a strain of 98 and 446. A Pearson coefficient close to 1 in samples ε_eq_98-I and ε_eq_98-II confirms the visible non-random Co distribution at that strain. In contrast, the much lower μ for sample ε_eq_446 indicates its more random Co distribution.

The 2D composition maps, which are additionally displayed in Figs. 11 and 12(a) show the distribution of the Co concentration in the same region of interest illustrated in the 3D reconstructed sub-volumes. The concentration is given in at.%, which is indicated according to color (15 = blue; 35 = red). As already expected from the experimental distributions, the composition map at a strain of 98 (Fig. 11(a)) show strong phase separation into Co-enriched areas. In between, the Co concentration is fairly high, but areas with very low Co concentration (< 15 at.%) are also visible. In Fig. 12(a), a large Cu-rich area is apparent in the center of the map although Co enriched areas are also present. The composition map obtained at a strain of 446 display distinct Co-rich areas as well, but regions between this areas show a more homogeneous Co distribution (Fig. 11(b)). Only small areas exhibit a Co concentration below 15 at.%.

**Fig. 13:**
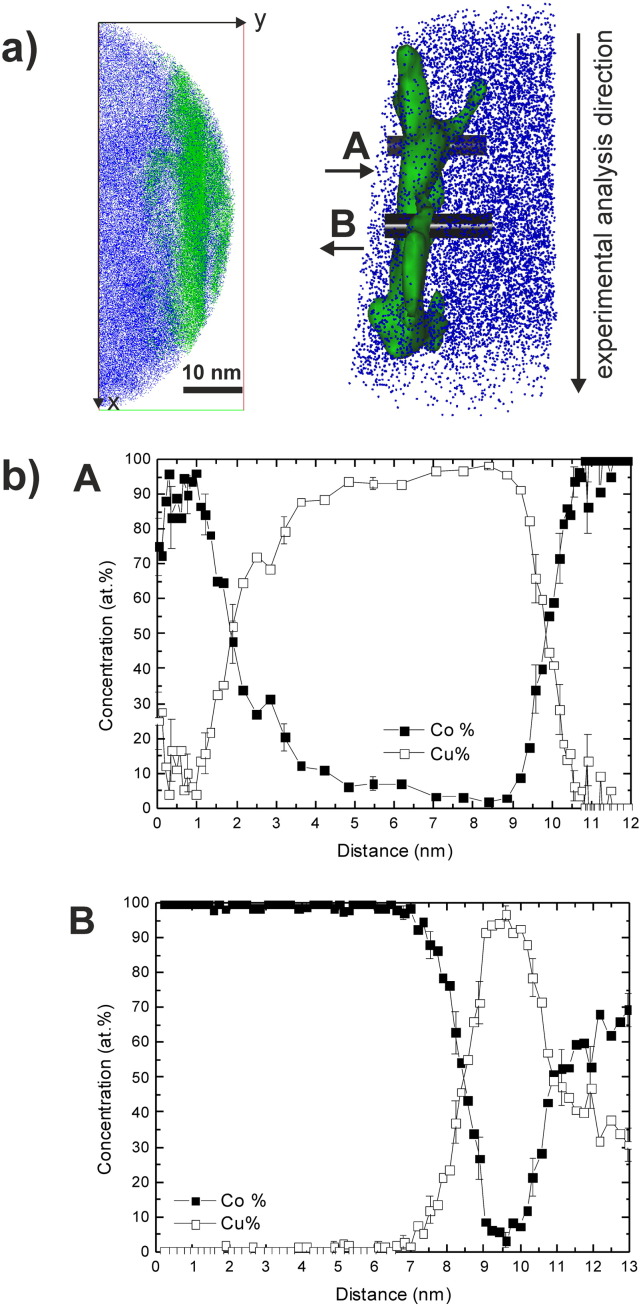
Cross section of a 3D reconstructed volume of the Cu–Co alloy sample deformed to a strain of 98. Cu atoms are displayed in green and Co atoms in blue. Two regions-of-interest (A and B) are selected. They are marked in the 3D reconstruction on the right, in which Co atoms are plotted in blue. The green isodensity surface corresponds to a Cu density of 31 Cu atoms nm^− 3^. Concentration profiles computed across A and B. Size of sampling cylinder A: Ø 2.5 nm × 12 nm, size of sampling cylinder B: Ø 3.0 nm × 13 nm. Thickness of sampling volume in A and B: 0.2 nm.

The bulk composition determined from the APT specimen prepared from the Co particle (ε_eq_98-3) is 97.2 ± 0.1 at.% Co and 2.7 ± 0.1 at.% Cu (Supplementary Fig. S1). In a small volume of the reconstruction, an interesting feature, Cu lamellae in the Co phase, are detected. The lamellae are very thin (only a few nanometers) and exhibit a non-planar morphology. In Fig. 13(a), a cross-section and a 3D reconstructed sub-volume containing the Cu lamellae are shown. The green isodensity surface in the 3D reconstructed sub-volume illustrates the lamellae and corresponds to a Cu density of 31 Cu atoms nm^− 3^. In Fig. 13(b), 1D-concentration profiles obtained from the small insets A and B are plotted (the analysis direction is indicated by the arrow in the 3D reconstructed sub-volume). The Co and Cu phases exhibit different evaporation fields, therefore a different electric field is required to induce field evaporation [33,34]. Hence, a valid concern is trajectory overlap in the APT reconstructions due to a local magnification effect, which can affect the reconstructed interface and therefore the 1D-concentration profiles in terms of width and concentration. The theoretical evaporation field of Cu (30 V nm^− 1^) is smaller compared to Co (36 V nm^− 1^) [20], which is the matrix in this case. Hence, the atomic density within the Cu lamellae is higher. In Ref. [35], it is proposed that the distance in the 1D-concentration profiles should be scaled based on the square root of the atomic density measured within each interval of the profile (i.e. bin size) to minimize density fluctuation effects. This correction to 1D-concentration profiles is valid if the interface is approximately along the experimental analysis direction (i.e. the z-direction of the reconstruction), which is the case in specimen (ε_eq_98-III). For the 1D-concentration profiles shown in Fig. 13(b), the proposed correction of the depth coordinates was applied. In both 1-D concentration profiles, only small amounts of Cu can be detected in the Co phase (or the Co particle). The concentration profile along B does not even contain any detectable amount of Cu in the Co phase. The thickness of the Cu lamella is below 10 nm in concentration profile A. It seems to be nearly unaffected by mechanical mixing as well as it contains only small amounts of Co (about 5–10 at.%). A concentration gradient appears on the left side of the Cu lamella, which is followed by a Co phase region containing some amount of Cu (up to 30 at.%). Such a mixed region, containing significant amount of both phases is also found next to the Cu lamellae in the concentration profile along B. The width of the Cu lamella is significantly smaller (< 3 nm) in this case. The roughness (or waviness) of the interface and its small width make it difficult to interpret the concentration gradient in profile A. Nevertheless, the width of the interface (~ 2 nm) next to the Co phase is similar in both cases.

## 4 Discussion

An ultrafine grain structure in the Cu matrix quickly develops similar as in the pure metals in the very early stages of HPT deformation [36]. A saturation of the hardness and refinement in the Cu matrix is reached at a strain of about 10. The grain size in this strain regime is significantly smaller compared to pure, bulk Cu deformed by HPT [37], but comparable to the steady state microstructure of pure HPT deformed powder Cu (microhardness of 207 ± 3.5 HV_0.5_, grain size 168 ± 10 nm).

**Fig. 14:**
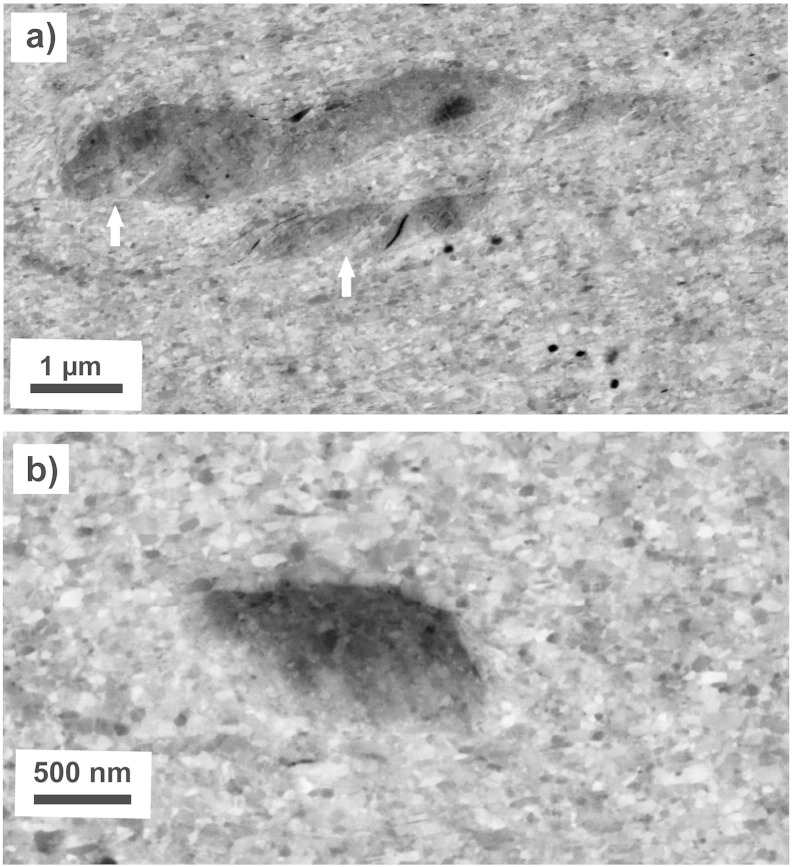
Back-scattered electron micrographs of Co particles in the Cu matrix at an equivalent strain ε_eq_ of 98 (a) and 446 (b).

The Cu–Co composite material consists of a softer, plastically deformable Cu phase with hard Co particles as second phase. The grain size of the Co phase is on the average below 100 nm in the initial state. From the view of the deformation process, Cu and Co exhibit also different crystal structures (Cu with a *fcc* structure and Co with a *hcp* structure). Hence, the imposed plastic strain is not distributed equally between the phases due to its different flow stresses and work hardening behavior resulting in complicated flow patterns and local stress concentrations [38]. The Cu matrix accommodates the major part of the applied strain, which implies that the Cu matrix has to undergo additional deformation to accommodate the harder Co phase. Load transfer to the Co phase can be either accommodated by plastic flow or fracture. “Large” Co particles (length > 1 μm) are slightly deformed in the beginning of the deformation indicated by their length increase (Fig. 6(c)). However, the length of the large Co particles continuously decreases at larger strains and the total number of particles per area exhibiting a size larger than 1 μm is continuously decreasing as well (Table 2). The microstructural refinement progresses slowly since the Co particles are mainly fragmented and fractured. An example illustrating the fragmentation and deformation behavior of the Co particles in the Cu matrix at medium deformation strain is shown in Fig. 14(a). The Co particles are steadily flattened and slightly elongated (like the large particle in the micrograph), fractured (marked in the micrograph) and rebound during continuing deformation similar as in ball milling or surface severe plastic deformation [39]. The same observations of the structural evolution of the Co particles have been made in the EFTEM investigations (Fig. 9).

In Ref. [6], it is shown that the fragmentation process of W particles during HPT deformation of a Cu–25%W composite material can be described by a fractal distribution. The fractal distribution (power law) model is frequently used to describe fragmentation processes in earth sciences [40]. According to

(2) $$N\;\left(>r\right)=C\;r^{-D}$$

the fractal dimensions D of N fragments with a size larger than r can be calculated. In Eq. (2), C is a constant. At low and medium strains, the fragmentation process of large Co particles in the Cu matrix can be described by the fractal distribution as well. Calculating the fractal dimensions from the plot shown in Fig. 6(b) results in similar fractal dimensions of D = 2.37, D = 2.51, D = 2.78, D = 3.28, D = 2.75 and D = 2.18 at strains of ε_eq_ ~ 0, ε_eq_ = 44, ε_eq_ = 88, ε_eq_ = 102, ε_eq_ = 167 and ε_eq_ = 209, respectively. For a higher strain (ε_eq_ = 446), the particle size distribution deviates from the fractal distribution model due to the low amount of detected particles and very small particle sizes (Table 2). Particles with a very small size (< 0.5 μm) still exist in the material, which are not detected by the image analysis software.

The saturation in the structural evolution in the Cu matrix and the slow progress of fragmentation of Co particles induce the relatively constant microhardness between strains of 10–70 (Fig. 2(b)). While the microhardness of the Cu–Co composite does not change at medium equivalent strains, the hardness increases linearly at higher equivalent strains (70 < ε_eq_ < 140). The hardness change is consistent with the microstructural evolution, in which a significant further grain size reduction in the Cu matrix is observable. The area fraction of the Co particles decreases from 18 to 3% in this strain regime. Larger Co particles seem to fracture more rapidly and the aspect ratio of the Co particles is quickly reduced from 4.8 to 3.1. Hence, even large Co particles are now approaching more and more a circular shape. The reason for the faster progressive fragmentation of the Co particles between 70 < ε_eq_ < 140 is the homogenization of the hardness of the Cu and Co phase in this strain regime. Although high hardness differences are recorded in the beginning of the deformation, the hardness ratio between the lowest and the highest measured hardness is quickly reduced (Fig. 4). Simultaneously, the grain size of the Cu matrix is further reduced (Fig. 7).

This raises the question what causes the refinement of the Cu phase even after its saturation grain size is already reached? The obtainable minimum grain size in the steady state during HPT deformation of pure metals is on the one hand material dependent, but it can be furthermore adjusted by alloying. Increasing the amount of alloying in single phase materials, decrease the steady state grain size during HPT [36]. APT measurements revealed that supersaturated solid solutions of about 24 at.% and 26 at.% Co in *fcc* Cu are achieved by HPT to a strain of 98 and 446 in homogeneous regions of the Cu matrix, respectively (Fig. 11(a) and (b)), which stabilizes the smaller grain size of the Cu matrix in the composite material compared to its pure counterpart.

The length of the small particles (size < 1 μm) is nearly unchanged during deformation, but their number is decreasing as well during deformation (Table 2). At a strain of 446, only 0.01 Co particles per μm^2^ in total are resolved in the back-scattered electron micrographs (an example is illustrated in Fig. 14(b)). It should be noted that no extensive deformation of the small particles (length < 1 μm) or fracture occurs during HPT deformation as not only their length but also the aspect ratio remains the same as a function of the equivalent strain (Fig. 6(c) and (d)). Particles with a circular shape are very stable and high strains are necessary for plastic deformation. In Ref. [41] it is shown that the high hydrostatic pressure suppresses any plastic deformation of small, hard particles even at high strains. Further fragmentation by fracturing becomes difficult for small particles as the probability of defects within particles (and in consequence their fracture probability) decreases with particle size [6,42,43]. But it is evident that a major part of even the smallest particles must be refined during deformation. In Ref. [16], dislocation motion and subsequent shearing and cutting of Co particles is proposed as a mechanism of further refinement of Co particles during ball milling. A necessary condition is the transformation from *hcp* Co to *fcc* Co as it is reported in Ref. [16]. Shearing by dislocation motion and cutting of particles is not supposed to be the mechanism of Co particle refinement during HPT deformation, because no transformation of *hcp* Co into the *fcc* structure is observed. The crystal structure of the Co particles is *hcp* even after strain of 100 is applied (Fig. 8(b)). Therefore, it is assumed that an “erosion process”, as already proposed in Ref. [5] for metal matrix composites, is responsible for the dissolution of the single Co particles, which are embedded in the Cu matrix. This is supported by the fact that small Co particles can be found at each deformation strain. It is furthermore supposed that the erosion process of small Co particles starts right from the beginning of the deformation. This is evident from the inhomogeneously refined microstructure of the Cu matrix at lower strains, in which regions with smaller grains are visible next to regions with larger grains (Fig. 7(a)–(d)).

Furthermore, APT measurements at two different strains reveal that the formed solid solutions are not homogeneous. The Co concentration is locally enriched as shown in 2D composition maps and experimental frequency distributions (Fig. 11(a) and (b)). Nevertheless, a more homogeneous distribution can be found for higher strains. In addition, APT and analytical TEM investigations at a strain of 98 show that the formation of supersaturated solid solutions in Co is not observed. Nearly no Cu is detected in the Co phase (Figs. 10 and 13). In close proximity to Co interfaces (Figs. 10 and 12(a)), the Co concentration in *fcc* Cu is smaller. Since the Cu–Co system has a positive heat of mixing, diffusion reactions lead to phase decomposition which is enhanced near Cu–Co interfaces.

Phase transformation of *hcp* Co to *fcc* Co, promoting the dissolution of Co particles, cannot be entirely excluded at this point, especially for particles having a size smaller than a few nm. During HPT deformation of pure Co powders, *hcp* to *fcc* phase transformation with some nanograins with other crystal structures (*hcp* Co and distorted *hcp* Co) are observed [44]. Further investigations about the exact mechanism behind the dissolution of the Co particles are certainly of interest and are currently being conducted.

## 5 Conclusion

SEM and hardness measurements combined with state-of-the-art nanoscale characterization methods were conducted to clarify structural evolution and phase formations in immiscible composites deformed by HPT on all length scales using the example of a simple Cu–Co model material. The findings can be summarized as follows:

(i) Supersaturated solid solutions up to 26 at.% Co in a *fcc* Cu–26 at.% Co alloy were obtained by HPT, contrary to the Co phase, which is not affected by Cu.
(ii) Although Co has an extended solubility in Cu, the formed supersaturated solid solutions are not homogeneous. Small Co particles remain in the Cu matrix and Co is still enriched in nanometer-sized clusters even at the highest amount of investigated strain.
(iii) The fragmentation process of large Co particles follows a fractal distribution at low and medium strains.
(iv) Dissolution of small Co particles starts at low strains. Further grain size refinement due to alloying induces a higher hardness of the Cu matrix, which accelerates the fragmentation and fracturing of large Co particles and promotes the dissolution of small Co particles at medium deformation strains.
(v) Shear induced mixing is suppressed by localized deformation of the ductile-brittle components and inhibition of dislocation transfer across *fcc*–*hcp* interfaces in this model system.

## Figures and Tables

**Table 1:**
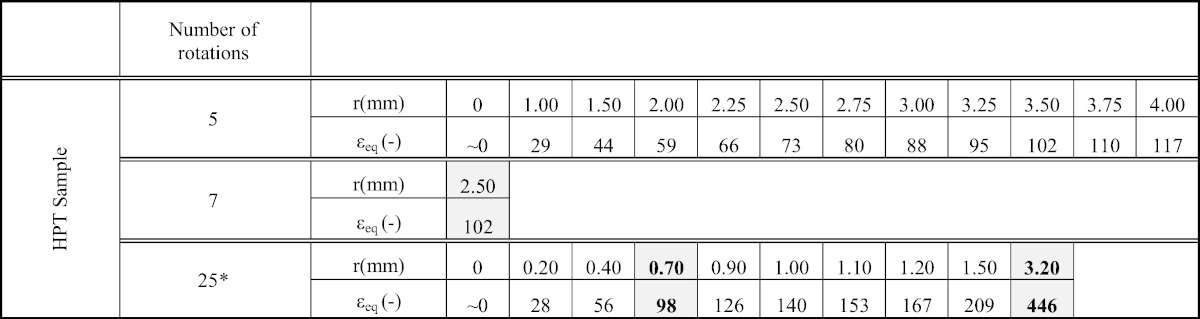
Overview of investigated positions (i.e. deformation strains) for different HPT samples (N = 5, 7, 25). At positions highlighted in gray TEM specimens have been prepared. At positions shown in bold, APT specimens have additionally been prepared. Nanoindentation measurements have been only performed on the HPT sample with 25 rotations.

⁎ SEM micrographs and nanoindentation measurements are only performed on HPT sample with 25 rotations.

**Table 2:** Overview of the total number of Co particles, the number of Co particles with a particle length > 1 μm and the number of Co particles with a particle length of < 1 μm given per area (μm^2^) at each deformation strain.

ε_eq_ (–)	~ 0	44	59	66	73	80	88	95	102	110	126	140	153	167	209	446
Total	0.169	0.114	0.130	0.101	0.100	0.110	0.117	0.132	0.139	0.130	0.081	0.032	0.019	0.028	0.014	0.008
Length > 1 μm	0.135	0.109	0.125	0.098	0.096	0.107	0.113	0.129	0.136	0.127	0.062	0.028	0.015	0.024	0.013	0.001
Length < 1 μm	0.033	0.005	0.005	0.004	0.004	0.004	0.005	0.003	0.003	0.003	0.019	0.004	0.003	0.004	0.001	0.007

## Appendix A Supplementary material

Supplementary Fig. S1 SEM micrograph of the Cu-Co alloy sample deformed to a strain of 98 showing the approximate position of the site specific APT specimens (ε_eq_98-I, ε_eq_98-II and ε_eq_98-III).
