# Supplementary figures and images for: Structural evolution and strain induced mixing in Cu–Co composites studied by transmission electron microscopy and atom probe tomography
Source: Mater Charact. 2015 Feb;100:178–91. doi: 10.1016/j.matchar.2014.12.022 (PMC4600609; doi:10.1016/j.matchar.2014.12.022)

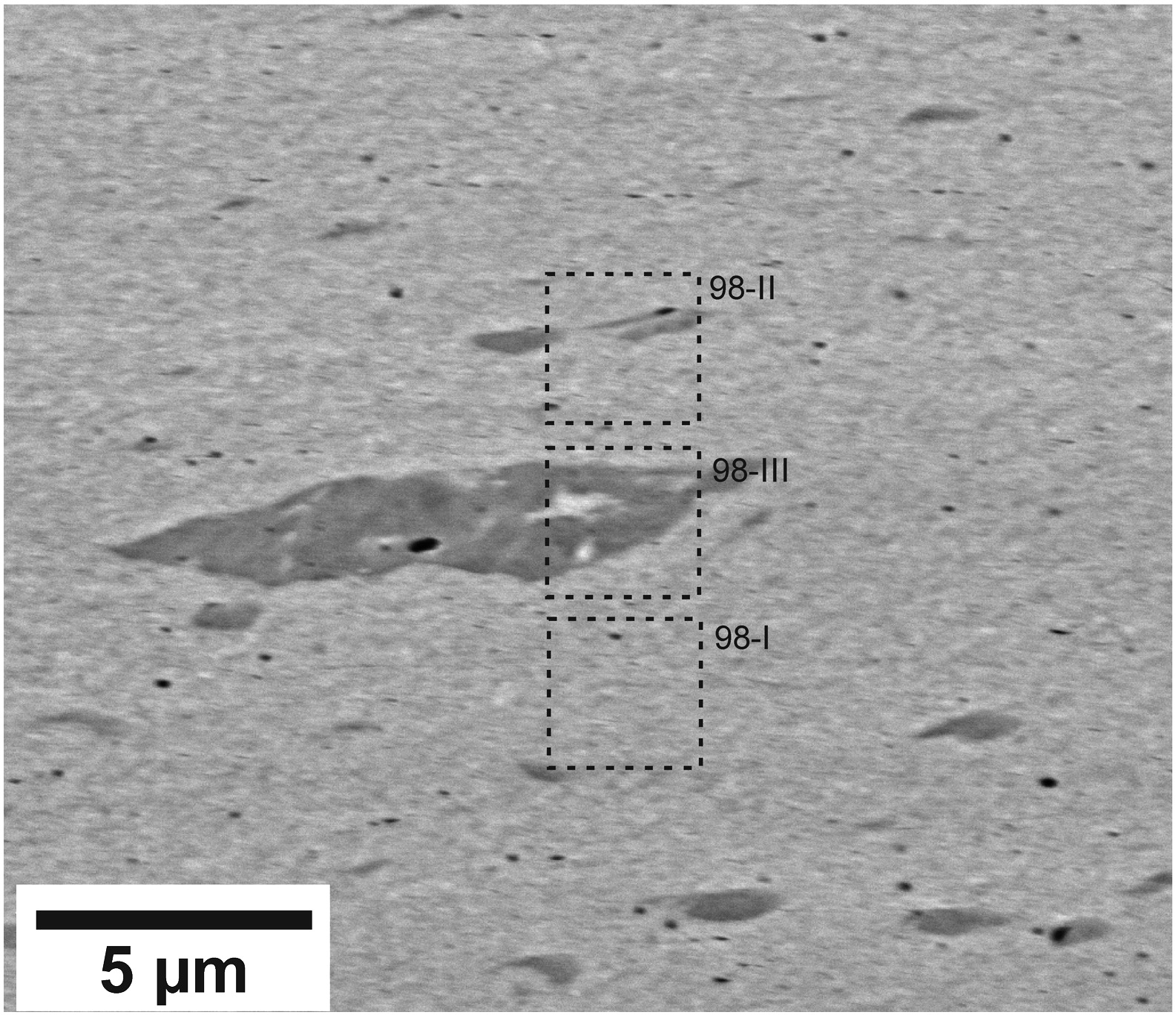

Supplement: Supplementary Fig. S1 — SEM micrograph of the Cu-Co alloy sample deformed to a strain of 98 showing the approximate position of the site specific APT specimens (εeq98-I, εeq98-II and εeq98-III). [file gr15.jpg]
